# Evaluating the Effect of Diagnosis–Intervention Packet (DIP) Reform in China on Hospitalization Outcomes for Patients with Chronic Obstructive Pulmonary Disease with Special Reference to M City

**DOI:** 10.3390/healthcare14091127

**Published:** 2026-04-22

**Authors:** Yile Li, Yingying Tao, Luyu Mo, Dan Wu, Chengcheng Li, Xuehui Meng

**Affiliations:** School of Humanities and Management, Zhejiang Chinese Medical University, Hangzhou 311402, China; 202312220701029@zcmu.edu.cn (Y.L.); 202421114611007@zcmu.edu.cn (Y.T.); 202521114611003@zcmu.edu.cn (L.M.); 20221020@zcmu.edu.cn (D.W.)

**Keywords:** DIP payment reform, COPD, hospitalization costs, hospital days, individual out-of-pocket payments

## Abstract

Background: Chronic Obstructive Pulmonary Disease (COPD) poses a substantial public health challenge in China owing to its increasing prevalence and substantial economic burden. In response, the diagnosis–intervention packet (DIP) payment reform was implemented to control healthcare costs and enhance service efficiency. Methods: To evaluate the effect of the DIP reform on medical costs, hospitalization days, and individual out-of-pocket payments for COPD inpatients in M City, a pilot city in central China, we conducted an interrupted time series (ITS) analysis using monthly reimbursement records from January 2020 to December 2023. The study included 84,410 hospitalized patients from a city-wide database of 3,241,233 inpatient records with COPD who met the inclusion criteria. The analysis focused on the total healthcare costs, length of stay, and individual out-of-pocket costs. Results: The DIP reform resulted in a 3.7% reduction (95% CI: 0.9% to 6.5%) in the total hospitalization costs in the first month post-reform, with a sustained monthly decline of 0.8% (95% CI: 0.5% to 1.1%). The length of stay decreased from 9.53 (95% CI: 9.31 to 9.75) to 8.74 days (95% CI: 8.62 to 8.86). Conversely, the proportion of out-of-pocket payments relative to total costs increased. Conclusions: While the DIP reform effectively reduced hospitalization costs and days, it led to an increase in individual out-of-pocket payments. Future research should focus on optimizing payment rules, enhancing the supervision of medical services, and refining health insurance policies to achieve the reform’s objectives better and alleviate the financial burden on patients.

## 1. Introduction

COPD is one of the most common chronic respiratory diseases worldwide, with high morbidity and mortality rates, and poses a major challenge to public health [1,2]. The high incidence of COPD not only significantly impairs the quality of life of the patients but also leads to a huge economic burden. According to the World Health Organization (WHO), COPD is the third leading cause of death worldwide. After stroke and ischemic heart disease, the high morbidity and mortality associated with COPD make it a critical public health priority [1,2].

In China, with the aging of the population and lifestyle changes, the incidence and mortality of COPD are on the rise year by year, with a prevalence rate of up to 13.7% among people aged 40 years and older and an estimated number of patients approaching 100 million. This not only imposes a significant financial burden on patients but also presents a substantial challenge to the healthcare insurance system [3,4,5]. To address the challenge of rapid growth in medical costs, the Chinese government has actively promoted the reform of health insurance payment methods in recent years to improve the efficiency and quality of medical services and contain the rapid growth of medical expenditures. In 2020, the Chinese government initiated the diagnosis–intervention packet (DIP) payment method under the framework of regional budgets. Reform [6,7,8]. DIP, as an innovative health insurance payment method, aims to control the unreasonable growth of healthcare costs by optimizing the allocation of healthcare resources and improving the efficiency of healthcare services. The core of the DIP payment method is to classify patients into different disease groups based on their disease diagnosis and treatment modalities and to set a payment standard for each disease group [9]. This payment method can not only incentivize medical institutions to reasonably control medical costs but also promote the standardization of medical services [10,11]. Prior to the reform, hospitals were reimbursed under fee-for-service (FFS), which rewarded volume [12]. Since January 2021, M City has implemented DIP, a prospective case-based payment system. Under DIP, each inpatient case is classified into a group based on its primary diagnosis and principal procedure [13,14]. Each group carries a pre-determined weight (or ‘score’) derived from historical cost data, reflecting the average expected resource consumption [12,13,14]. High-scoring illnesses are complex, resource-intensive cases; low-scoring illnesses are routine, less costly ones [13]. A hospital’s total reimbursement is calculated by multiplying the sum of scores for all its treated cases by a region-wide monetary conversion rate, which is set annually by dividing the total regional budget by the sum of all hospitals’ scores [12]. Thus, DIP creates a ‘surplus retention, loss absorption’ incentive: hospitals profit from efficiency gains but absorb losses from cost overruns [13].

This encourages efficiency (e.g., reducing length of stay, eliminating unnecessary services) but also carries risks of strategic behaviors such as up-coding or patient selection—underscoring the need to monitor quality alongside costs [12,13,14]. This need for quality monitoring is closely tied to the governance framework within which DIP operates. The DIP system’s effectiveness depends critically on the governance framework established by national and local health authorities.

At the national level, the National Healthcare Security Administration (NHSA) provides the overarching regulatory architecture, including standardized disease grouping guidelines, budget control principles, and technical parameters for payment calculation [15]. This central steering ensures consistency and facilitates cross-regional learning. At the local level, municipal health security bureaus adapt these national guidelines to their specific contexts, setting payment coefficients that reflect hospital characteristics and regional disease profiles [16]. The performance of fund transfer—whether it successfully contains costs while maintaining quality—is, therefore, shaped by the quality of local governance, including the accuracy of budget allocation, the effectiveness of supervision mechanisms, and the capacity to negotiate with healthcare providers [17]. Conversely, weak governance—such as insufficient monitoring or delayed settlement—may undermine reform objectives, potentially leading to strategic provider behaviors like up-coding or patient selection. Thus, DIP reform is not merely a technical payment mechanism but a complex governance intervention that requires robust institutional capacity at both national and local levels to achieve its intended outcomes [18].

However, the treatment of COPD patients is complex, and the hospitalization cost structure and adaptability of the health insurance payment method reform still need to be studied in depth [19,20]. The hospitalization cost of COPD patients usually includes the medical cost, the number of days of hospitalization, and the cost of drugs. The distribution and changing trend of these costs may be affected by a variety of factors, such as the severity of the patient’s condition, choice of treatment plan, and management level of the medical institution [21]. In addition, there is uncertainty about the effect of the DIP payment reform on the healthcare costs of hospitalized patients with COPD. On the one hand, the DIP payment method may reduce patients’ medical costs by incentivizing healthcare institutions to optimize treatment plans and reduce unnecessary medical interventions; on the other hand, the DIP payment method may also have an effect on healthcare institutions’ healthcare service behaviors, such as inducing healthcare institutions to choose high-scoring illnesses or to reduce the admission and treatment of low-scoring illnesses [22,23].

DIP reform influences hospitalization outcomes by altering provider incentives. The shift from fee-for-service to prospective case-based payment encourages hospitals to reduce length of stay and eliminate unnecessary services, thereby lowering total costs. However, these cost-containment pressures may inadvertently increase patient out-of-pocket payments, for instance, through shifts to non-covered services or patient selection. Thus, evaluating DIP requires simultaneous examination of total costs, length of stay, and out-of-pocket payments—the three indicators in this study—as they collectively capture both intended efficiency gains and potential unintended consequences.

To provide a robust theoretical foundation for our analysis, we draw upon established frameworks in health services research. Andersen’s Behavioral Model of Health Services Use conceptualizes how predisposing characteristics (e.g., age, sex), enabling resources (e.g., health insurance type, hospital level), and need factors (e.g., disease severity, comorbidities) influence healthcare utilization and outcomes [24]. Donabedian’s Structure–Process–Outcome Model further elucidates how structural components of the healthcare system (e.g., hospital governance, payment mechanisms) shape care processes (e.g., treatment patterns, length of stay) and ultimately patient outcomes (e.g., costs, readmissions, mortality) [25]. In the context of DIP reform, these frameworks guide our selection of control variables and help conceptualize the pathways through which payment changes may affect hospitalization outcomes. While our administrative data do not capture all clinically relevant factors (such as detailed comorbidity profiles or disease severity measures), we employ available variables—age, sex, hospital level, and insurance type—as proxies for key Andersen model constructs, and explicitly acknowledge the limitations arising from unmeasured confounders.

This study assesses the DIP reform’s effect on COPD inpatient outcomes using data from M City, a pilot city in central China. As one of the 71 pilot cities nationally, M City has achieved full coverage of the DIP payment method [26]. This study makes two key contributions to the literature. First, it provides the first empirical assessment of the differential effects of DIP reform across hospital levels (primary, secondary, and tertiary) and health insurance types (URRMI and UEBMI), offering granular insights for policy refinement. Second, using interrupted time series (ITS) analysis—a rigorous quasi-experimental method—we evaluate both the immediate and sustained effects of the reform on medical costs, hospitalization days, and individual out-of-pocket payments. The findings provide empirical evidence for health insurance payment reform and policy recommendations for optimizing medical services for COPD patients [27].

## 2. Data Sources and Methods

### 2.1. Study Setting and Population

M City, a prefecture-level city in Hubei Province, China, served as the study setting. As of 2024, the city’s resident population reached 3,153,200, with a per capita GDP of 73,489.10 yuan, representing a medium level of economic and social development. M City was among the first 71 pilot cities nationally to implement the DIP payment reform. Within Hubei Province, M City pioneered the reform, with 5393 core diseases and 928 comprehensive diseases in its DIP disease catalog. The reform achieved comprehensive coverage across all healthcare organizations, with basic health insurance covering 98% of the city’s population. In special cases involving innovative technologies or critical illnesses resulting in high costs, the health insurance department reimburses reasonable hospitalization expenses from the global budget. The reform also incorporates payment coefficients tailored to the specialized characteristics, hospital grades, and functional positioning of each institution, with dynamic adjustments implemented over time.

We obtained monthly reimbursement records from the Hubei Provincial Medical Insurance Information Platform (January 2020–December 2023). Each record included patient age, sex, disease diagnosis (ICD-10 codes), hospital level, type of health insurance, dates of admission and discharge, and detailed cost components. Hospital levels in China are classified as primary, secondary, and tertiary, and the two main types of health insurance are Urban and Rural Residents’ Basic Medical Insurance (URRMI) and Urban Employee Basic Medical Insurance (UEBMI).

**Figure 1:**
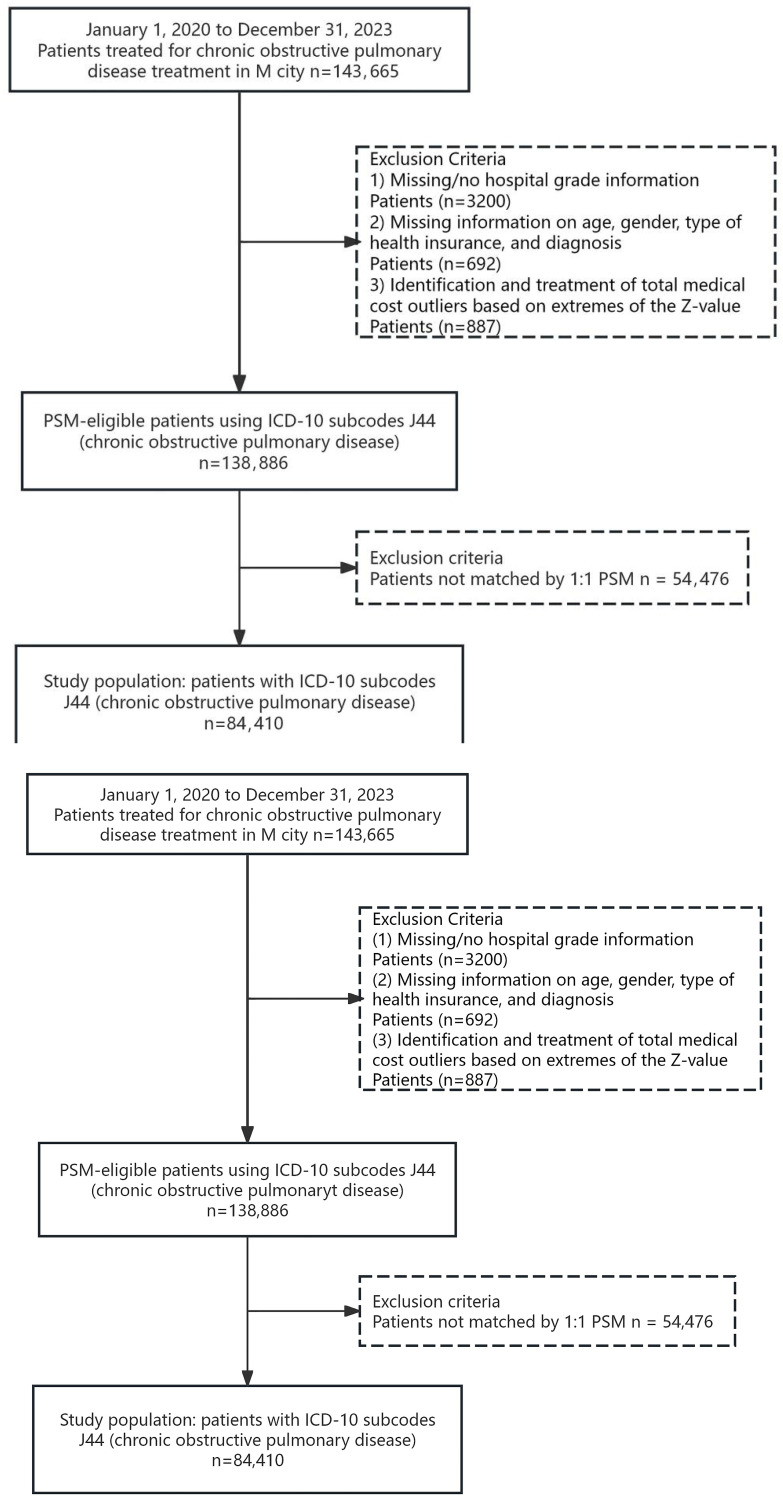
Specific selection and exclusion process of study participants.

The initial dataset comprised 143,665 inpatient records with a primary diagnosis of COPD. During data preprocessing, we employed the WPS 2021 software to remove records with missing information on hospital grade, age, sex, insurance type, or diagnosis. Outliers in total medical expenses were identified using the Z-score extreme value method (|Z| > 3) in SPSS 27 and subsequently excluded. After these steps, 138,886 records remained eligible for further analysis. The detailed screening and exclusion process is illustrated in Figure 1. Ethical approval was granted by the authors’ university (approval number: 20240516-3), and the requirement for individual informed consent was waived due to the retrospective nature of the study.

#### 2.1.1. Hospital Level

There are three levels of hospitals in China: level I, level II, and level III. Level I hospitals are primary healthcare institutions that provide comprehensive services such as prevention, medical care, and rehabilitation directly to community residents, and generally have a bed capacity of approximately 100. Level II hospitals are local hospitals that provide medical and healthcare services to multiple communities, mainly treating common diseases and high morbidity, with a bed capacity of approximately 300. Tertiary hospitals are municipal medical and preventive technology centers with comprehensive medical treatment, teaching, and research capabilities, mainly treating difficult and complicated diseases and critically ill patients, with a bed capacity of approximately 500.

#### 2.1.2. Types of Health Insurance

China’s medical insurance system is divided into two main categories according to the scope of services and the population covered: urban and rural residents’ medical insurance (URRMI) and urban employees’ medical insurance (UEBMI). URRMI is mainly for rural residents and urban non-employed residents, and is led by the government, with the government and individuals jointly contributing to the cost of insurance, aiming to provide basic medical protection, while UEBMI is mainly for urban employed persons, with the unit and individuals jointly contributing to the cost of insurance to protect their basic medical needs. In this study, two types of URRMI and UEBMI were selected for research, and these two types of medical insurance together constitute China’s multilevel medical insurance system.

#### 2.1.3. Chronic Obstructive Pulmonary Disease (COPD)

The prevalence of COPD in China and progress in its prevention and treatment are of great public health significance. According to the latest research data, the prevalence of COPD in Chinese adults aged 20 years and older is 8.6%, and the prevalence in people aged 40 years and older is as high as 13.7%, with an estimated number of patients of nearly 100 million, making it the third leading cause of death in China after stroke and ischemic heart disease. By screening 3,241,233 participants enrolled in City M, we screened a total of 84,410 COPD patients from January 2020 to December 2023 for analysis.

### 2.2. Study Design

This study employed an interrupted time series (ITS) design to evaluate the DIP reform’s effect on hospitalization outcomes for COPD patients. ITS is a quasi-experimental approach widely used to assess the effect of population-level interventions when a control group is unavailable or when randomization is not feasible [28,29]. By analyzing data at multiple time points before and after an intervention, ITS can distinguish the intervention’s effect from underlying secular trends, thereby supporting inference in observational settings [30]. In this study, January 2021 was designated as the intervention point, as M City implemented the DIP reform universally and simultaneously across all hospitals on this date. The observation period spanned 48 months (January 2020 to December 2023), including 12 pre-intervention and 36 post-intervention months. This length of observation provides sufficient statistical power to detect both immediate (step) changes and sustained (slope) effects of the reform, following established methodological recommendations.

### 2.3. Sampling and Matching

To ensure comparability and minimize selection bias, we employed propensity score matching (PSM) between the pre-reform (2020) and post-reform (2021–2023) groups. PSM is a widely used method to balance observed covariates between treated and untreated groups, thereby approximating the conditions of a randomized experiment and reducing confounding in non-randomized settings [31,32]. We performed annual 1:1 nearest neighbor matching without replacement using a caliper of 0.01. Age and sex were selected as matching covariates. Age is a central determinant of COPD outcomes and costs, as disease severity, comorbidity burden, and healthcare utilization increase progressively with age [31]. Sex was included for two reasons: first, a growing body of evidence documents sex-based differences in COPD presentation, symptom burden, exacerbation risk, and healthcare-seeking behavior [32,33]; second, baseline comparisons revealed a statistically significant difference in sex distribution between the pre-reform and post-reform groups prior to matching (*p* = 0.008), which warranted adjustment to avoid potential confounding.

Matching was conducted separately for each year (2020 matched with 2021, 2020 with 2022, and 2020 with 2023) to account for potential temporal changes in patient characteristics. After matching, the final analytic sample comprised 84,410 COPD inpatients. Balance diagnostics confirmed that standardized mean differences for all covariates were below the conventional threshold of 0.1, indicating successful covariate balance between groups [31]. The matched sample was then used as the basis for the interrupted time series analysis, ensuring that any observed changes following the reform are attributable to the policy itself rather than to pre-existing differences in patient characteristics between the pre- and post-reform periods.

### 2.4. Measured Variables

During the observation period, we selected the total healthcare costs, length of stay, and percentage of individual out-of-pocket costs for each patient at the time of discharge as indicators of the main effect. The length of stay (LOS) was calculated as the difference between discharge and admission dates. Given that LOS approximated a normal distribution, it was reported in its original metric (days) as mean (standard deviation) to characterize central tendency and dispersion. To eliminate the effect of inflation, all cost data involved in the study were adjusted according to the 2020 standards using the annual Chinese consumer price index, According to the National Bureau of Statistics of China, the annual Consumer Price Indices (CPIs) for 2020 through 2023 were 102.5, 100.9, 102.0, and 100.2, respectively (using previous year = 100 as base). Since the DIP reform was implemented at the end of 2020 and officially launched in January 2021, using 2020 as the cost reference standard allows for direct comparability of healthcare costs before and after the reform intervention. In addition, to correct for the skewed distribution of the data, we first logarithmized the total medical costs and out-of-pocket costs at the individual patient level. For length of stay (LOS), we did not apply a logarithmic transformation because its distribution, while moderately right-skewed, did not exhibit the extreme skewness characteristic of cost data, and the ITS model with robust standard errors adequately accounts for heteroscedasticity [33]. The monthly time-series for the ITS analysis was then constructed by calculating the average of these log-transformed values for all patients discharged within each month. The proportion of the costs borne by the individual was calculated by dividing the patient’s out-of-pocket portion by the total medical costs. We acknowledge that our data lack clinical severity measures (e.g., lung function, Charlson/Elixhauser comorbidity indices) and socioeconomic factors (e.g., education, income), which are not available in administrative claims and may confound estimates.

Given that healthcare cost and length of stay data are typically right-skewed—approximating gamma and negative binomial distributions, respectively—we employed log-transformation for costs in the ITS models and used robust standard errors to ensure valid inference despite distributional assumptions [33].

### 2.5. Statistical Analysis

For continuous data, we presented the data in the form of means (standard errors), while categorical data were expressed as percentages. Before and after the implementation of the DIP payment system reform in M, we conducted comparative analyses of the overall sample’s outcome variables and patient characteristics by hospital class, using t-tests and chi-square tests. In addition, we conducted an ITS analysis using segmented regression models. January 2021 was designated as the policy implementation node. The analysis utilized monthly aggregated data points over 48 months to assess the effect of the DIP policy. As described in the Measured Variables section, the outcome variables ($Y_{t}$) for total costs and out-of-pocket costs in the ITS model are the monthly averages of the log-transformed individual-level values. The model assumes that the implementation of the DIP policy will change patients’ total health care costs, length of stay, and individual payment percentage, and the ITS regression model constructed is shown below:

$$Y_{t}=\beta_{0}+\beta_{1}T_{t}+\beta_{2}DIP_{t}+\beta_{3}DIP_{t}T_{t}+\varepsilon_{t}$$

where $\beta_{0}$ is the estimated value of the baseline level of the outcome variable at the beginning of the study; $\beta_{1}$ is the monthly slope of the outcome variable before the DIP payment method reform [34]; $\beta_{2}$ represents the amount of instantaneous change in the outcome variable after the DIP payment method reform compared to the counterfactual outcome; $\beta_{3}$ is the change in trend of the outcome variable after the DIP payment method reform compared to the pre-reform period; and $\beta_{1}$ + $\beta_{3}$ is the trend after the reform. $Y_{t}$, on the other hand, represents the outcome variable for each month; $T_{t}$ is the time variable from the beginning of the study to the end of the study; $DIP_{t}T_{t}$ is the interaction term between time and the DIP intervention [35]; $\varepsilon_{t}$ is the estimation of the random error at time t; and $DIP_{t}$ is the policy intervention variable, operationalized as a dummy variable taking the value 0 for the pre-reform period (January 2020 to December 2020) and 1 for the post-reform period (January 2021 to December 2023).

To address autocorrelation in the ITS models, we used a systematic correction strategy guided by statistical diagnostics. Autocorrelation is a common concern in time-series data, as observations closer in time tend to be more similar, which can violate the independence assumption of ordinary least squares regression and lead to biased standard errors if left uncorrected. Initially, all models were fitted using ordinary least squares (OLSs). Subsequently, formal tests were conducted using commands to verify the presence and order of autocorrelation. Based on the diagnostic results: for models exhibiting first-order autocorrelation, we applied the Prais–Winston method, which is specifically designed to correct for AR(1) processes and has been shown to perform well in ITS applications in terms of mean square error and coverage [36]; for models with no autocorrelation, or with second-order and higher-order autocorrelation, we utilized the Newey–West method with an appropriately selected lag order to obtain heteroscedasticity- and autocorrelation-consistent (HAC) standard errors [37]. This dual approach ensures robust statistical inference across all model specifications. In addition, for the sensitivity analyses, we set a series of dummy points in time for the non-interference period to assess whether changes in the outcome variables could be attributed to the implementation of the DIP payment methodology reform [38,39].

To further address potential confounding from the COVID-19 pandemic, we note that the entire study period (January 2020 to December 2023) falls within the pandemic era, with no pre-pandemic baseline included. The pandemic’s impact on healthcare utilization was, therefore, relatively constant across the pre- and post-reform phases. Moreover, China’s COVID-19 containment policies remained relatively stable during 2021–2022, with major shifts occurring only in late 2022. Our sensitivity analyses, placing placebo intervention points at different times (e.g., mid-2020, mid-2022), revealed no significant structural breaks at those points, supporting the attribution of observed changes to the DIP reform rather than to unmeasured pandemic shocks.

First, we conducted an ITS analysis of patients discharged from all hospitals. We then conducted further exhaustive analyses of the effect of the DIP payment reform on all levels of hospitals (including primary, secondary, and tertiary) and types of health insurance (urban and rural residents’ health insurance and urban workers’ health insurance). We used 5% as the threshold for statistical significance, and all statistical analyses were conducted using STATA 17.0.

## 3. Results

### 3.1. Sample Characteristics and Total Hospitalization Costs

Our analysis included 15,234 and 69,175 insured discharges before and after the DIP payment reform, respectively. Table 1 shows the characteristics of the study sample. The mean age of patients before and after the reform was 68.51 and 69.33 years, respectively; males accounted for 76.4% of the total sample size, and about 86.8% of the insured patients were enrolled in the basic urban and rural residents’ health insurance.

There were six level III hospitals, 44 level II hospitals, and 214 level I hospitals, according to hospital level. Before the reform, level II hospitals had the highest percentage of discharged patients, accounting for 44.74% of the city’s discharges, with no significant difference from level I hospitals at 43.97%. After the reform, level I hospitals had the highest percentage of discharged patients, accounting for 48.55% of the city’s discharges. In addition, tertiary hospitals, which have always had the lowest percentage of discharged patients, saw their share of discharges fall by 0.44 percentage points after the DIP reform. In contrast, the share of tertiary hospitals increased by 4.58 percentage points.

Mean unadjusted expenditure per patient decreased from ¥6015 (95% CI: ¥5903 to ¥6127) before the reform to ¥5138 (95% CI: ¥5094 to ¥5182) after (*p* < 0.001). Average hospitalization days decreased from 9.53 days (95% CI: 9.44 to 9.62) to 8.74 days (95% CI: 8.68 to 8.80) (*p* < 0.001). The individual out-of-pocket payment ratio increased from 17.7% (95% CI: 17.4% to 18.0%) to 21.2% (95% CI: 20.9% to 21.5%), representing a 3.5 percentage point increase (95% CI: 3.2 to 3.8). The corresponding effect sizes were modest: Cohen’s d for total costs was 0.11, for length of stay was 0.15, and for the OOP ratio was 0.27. These small-to-modest magnitudes suggest that while the large sample size (n = 84,410) produced statistically significant findings, the practical significance of the observed changes should be interpreted with appropriate caution.

**Table 1:** Characteristics of the sample of insured patients in M City, 2020–2023.

Variables	Before DIP Reform, 2020	After DIP Reform, 2021–2023	*p*-Value
Discharge cases, No.	15,234	69,175	-
Age, mean (SD)	68.51 (10.05)	69.33 (9.71)	<0.001
Sex, No. (%)	-	-	0.008
Male	11,755 (77.16)	52,746 (76.25)	-
Female	3479 (22.84)	16,429 (23.75)	-
Medical insurance, No. (%)	-	-	<0.001
Urban and rural residents’ basic medical insurance	13,520 (88.75)	59,826 (86.49)	-
Urban employee basic medical insurance	1714 (11.25)	9349 (13.51)	-
Hospital level, No. (%)	-	-	<0.001
Tertiary (N = 6)	6699 (43.97)	33,586 (48.55)	-
Secondary (N = 44)	6816 (44.74)	28,090 (40.61)	-
Primary (N = 214)	1719 (11.28)	7499 (10.84)	-
Total expenditure per case, mean (SD), RMB	6015.00 (7012.89)	5137.80 (5769.06)	<0.001
Length of stay, mean (SD), d	9.53 (5.74)	8.74 (4.19)	<0.001
Individual out-of-pocket ratio (%), mean (SD)	17.67 (12.53)	21.18 (13.08)	<0.001
Individual out-of-pocket, mean (SD), RMB	1012.89 (1621.08)	1083.89 (1487.45)	<0.001

DIP indicates diagnosis–intervention packet payment reform; N is the number of hospitals. Total per-patient hospitalization costs were adjusted to 2020 using the annual Chinese consumer price index. In Table 2, ‘lg’ denotes the logarithmically transformed total expenditure per case using natural logarithms (ln). Values in parentheses represent standard deviations.

**Table 2:** Interrupted time series (ITS) analysis of total expenditures per case before and after diagnosis-intervention packet (DIP) payment reforms.

Ig (Total Expenditure per Case)	Baseline Monthly Slope (β1)	Step Change (β2)	Monthly Slope Change (β3)	Constant (β0)
	Estimate (95%CI)	Estimate (95%CI)	Estimate (95%CI)	Estimate (95%CI)
All hospitals	0.005 (0.003, 0.007) ***	−0.037 (−0.069, −0.009) *	−0.008 (−0.010, −0.005) ***	3.603 (3.587, 3.619) ***
Hospital level				
Tertiary hospitals	0.006 (0.004, 0.008) ***	−0.061 (−0.092, −0.030) ***	−0.007 (−0.010, −0.004) ***	3.321 (3.305, 3.336) ***
Secondary hospitals	0.007 (0.005, 0.009) ***	−0.039 (−0.059, −0.019) ***	−0.009 (−0.011, −0.007) ***	3.761 (3.537, 3.792) ***
Primary hospitals	0.000 (−0.008, 0.009)	−0.011 (−0.054, 0.028)	−0.011 (−0.023, 0.001)	4.007 (3.939, 4.075) ***
Medical insurance, No. (%)				
Urban and rural residents’ basic medical insurance	0.006 (0.004, 0.008) ***	−0.062 (−0.093, −0.031) ***	−0.007 (−0.009, −0.005) ***	3.321 (3.306, 3.335) ***
Urban employee basic medical insurance	0.015 (0.004, 0.025) **	−0.081 (−0.162, 0.000)	−0.020 (−0.030, −0.010) ***	3.338 (3.265, 3.411) ***

DIP indicates diagnosis–intervention packet payment reform; CI is confidence interval. To account for the effect of inflation, we adjusted total expenditures per patient case using the annual Chinese CPI with 2020 as the base year. In the model, total expenditures per case were log-transformed. We used the Newey–West method with the Prais–Winston method to adjust for autocorrelation with different lags, respectively. Values in parentheses represent standard deviations (Table 1) or 95% confidence intervals (Table 2, Table 3 and Table 4). * *p* < 0.05; ** *p* < 0.01; *** *p* < 0.001.

**Table 3:** Interrupted time series (ITS) analysis of length of stay (LOS) before and after diagnosis–intervention packet (DIP) payment reforms.

Length of Stay	Baseline Monthly Slope (β1)	Step Change (β2)	Monthly Slope Change (β3)	Constant (β0)
	Estimate (95%CI)	Estimate (95%CI)	Estimate (95%CI)	Estimate (95%CI)
All hospitals	0.063 (−0.010, 0.126)	−0.579 (−0.997, −0.161) **	−0.099 (−0.172, −0.026) **	9.530 (8.780, 9.805) ***
Hospital level				
Tertiary hospitals	0.063 (−0.018, 0.144)	−0.731 (−1.254, −0.209) **	−0.093 (−0.175, −0.012) *	2.264 (7.625, 8.902) ***
Secondary hospitals	0.071 (−0.008, 0.149)	−0.508 (−0.953, −0.063) *	−0.099 (−0.178, −0.020) *	9.852 (9.240, 10.464) ***
Primary hospitals	0.047 (−0.096, 0.189)	−0.506 (−1.310, 0.297)	−0.089 (−0.232, −0.054)	10.708 (9.667, 11.749) ***
Medical insurance, No. (%)				
Urban and rural residents basic medical insurance	0.066 (−0.009, 0.141)	−0.735 (−1.202, −0.268) **	−0.096 (−0.172, −0.020) *	8.214 (7.632, 8.797) ***
Urban employee basic medical insurance	−0.059 (−0.432, 0.314)	−0.883 (−2.960, 1.193) *	0.015 (−0.359, −0.388)	10.355 (7.289, 13.421) ***

DIP indicates Diagnosis–intervention packet payment reform; CI is confidence interval. We used the Newey–West method with the Prais–Winston method to adjust for autocorrelation with different lags, respectively. Values in parentheses represent standard deviations (Table 1) or 95% confidence intervals (Table 2, Table 3 and Table 4). * *p* < 0.05; ** *p* < 0.01; *** *p* < 0.001.

**Table 4:** ITS analysis of OOP before and after DIP payment reforms.

Individual Out-of-Pocket Ratio	Baseline Monthly Slope (β1)	Step Change (β2)	Monthly Slope Change (β3)	Constant (β0)
	Estimate (95%CI)	Estimate (95%CI)	Estimate (95%CI)	Estimate (95%CI)
All hospitals	−0.001 (−0.002, 0.001)	0.016 (0.000, 0.032) *	0.002 (0.000, 0.003) **	0.180 (0.170, 0.191) ***
Hospital level				
Tertiary hospitals	0.033 (−0.033, 0.100)	0.018 (0.001, 0.035) *	0.111 (0.018, 0.204) *	0.206 (0.196, 0.216) ***
Secondary hospitals	−0.078 (−0.207, 0.051)	0.020 (0.002, 0.038) ***	0.230 (0.083, 0.378) ***	0.185 (0.175, 0.195) ***
Primary hospitals	−0.375 (−0.672, −0.078) *	0.015 (−0.005, 0.035)	0.505 (0.202, 0.808) ***	0.165 (0.155,0.175) ***
Medical insurance, No. (%)				
Urban and rural residents’ basic medical insurance	0.000 (−0.000, 0.001)	0.013 (−0.004, 0.029)	0.001 (0.000, 0.002) *	0.113 (0.108, 0.118) ***
Urban employee basic medical insurance	−0.000 (−0.003, 0.003)	0.004 (−0.026, 0.034)	−0.002 (−0.005, 0.001)	0.227 (0.202, 0.253) ***

DIP indicates diagnosis–intervention packet payment reform; CI is confidence interval. We used the Newey–West method with the Prais–Winston method to adjust for autocorrelation with different lags, respectively. Values in parentheses represent standard deviations (Table 1) or 95% confidence intervals (Table 2, Table 3 and Table 4). * *p* < 0.05; ** *p* < 0.01; *** *p* < 0.001.

### 3.2. Total Expenditure per Case

**Figure 2:**
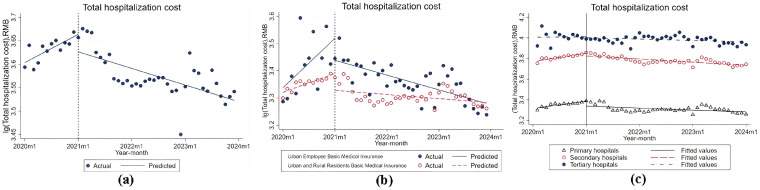
Total hospitalization cost. (**a**) Trends of actual and predicted total hospitalization costs; (**b**) Comparison of total hospitalization costs between Urban Employee Basic Medical Insurance and Urban and Rural Residents Basic Medical Insurance; (**c**) Trends of total hospitalization costs across primary, secondary, and tertiary hospitals.

Table 2 and Figure 2a present the baseline levels and trends of inflation-adjusted patient healthcare costs before and after the implementation of DIP reform. Before the reform, the total cost per case in all hospitals in M showed a significant increase of 0.5% per month (*p* < 0.001). In contrast, the total cost per case showed a significant 3.7% decrease (*p* < 0.05) in the first month after the DIP reform. A significant monthly downward trend (0.8%, *p* < 0.001) was observed after the reform progressed, which resulted in a sustained 0.3% decrease in total costs per month after the reform compared with the upward trend before the intervention (*p* < 0.001).

Subsequently, analysis by further disaggregation according to the type of health insurance (see Figure 2b) shows that urban and rural residents have higher baseline levels and cost changes before and after the reform than urban workers, although both show a general downward trend.

In addition, an analysis of the classification of hospitals by level (Figure 2c) shows that the total patient costs in hospitals I, II, and III show a broadly increasing and then decreasing trend after the policy reforms. Tertiary hospitals had the highest costs, followed by secondary hospitals, and primary hospitals had the lowest. Hospitalization costs at level III, level II, and level I hospitals showed a monthly downward trend after the reform.

These trends reflect the DIP reform’s effect on efficiency across insurance types and hospital levels.

### 3.3. Length of Stay

**Figure 3:**
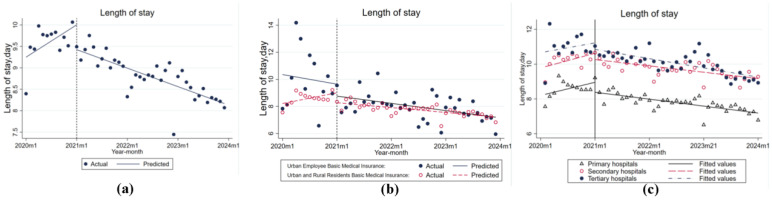
Length of stay. (**a**) Trends of actual and predicted length of stay; (**b**) Comparison of length of stay between Urban Employee Basic Medical Insurance and Urban and Rural Residents Basic Medical Insurance; (**c**) Trends of length of stay across primary, secondary, and tertiary hospitals.

By analyzing the trend of LOS changes before and after the implementation of the DIP reform for different types of health insurance and hospital levels, it was found that the average LOS per case in all hospitals in M City first increased and then decreased. As shown in Figure 3a, before the reform, the total number of hospitalization days showed an increasing trend of 0.063 days per month (*p* > 0.05), but the results were not significant. On the other hand, the first month after the reform showed a significant decrease in hospitalization days of 0.579 days (*p* < 0.01). As the reform policy progressed, a change of −0.099 (*p* < 0.01) occurred in the monthly slope compared with the pre-reform month.

Analyzing Table 3 and Figure 3b, the hospitalization days of urban and rural residents showed an overall trend of first increasing and then decreasing. Before the reform, the average number of hospitalization days for urban workers showed a monthly decline of 0.059 days (*p* > 0.05), while the monthly downward trend of hospitalization days slowed to 0.044 days (*p* > 0.05) after the policy intervention. The overall number of hospitalization days was higher for urban workers than for urban and rural residents.

In addition, by analyzing Figure 3c and further analyzing the indicator of hospital level, the average number of days of hospitalization at all levels of hospitals showed an upward and then a downward trend, and the average number of days of hospitalization was highest in level III hospitals and lowest in level I hospitals. After the reform, the number of hospitalization days in level I hospitals decreased at the fastest rate by 0.042 per month (*p* > 0.05).

### 3.4. Individual Deductibles

**Figure 4:**
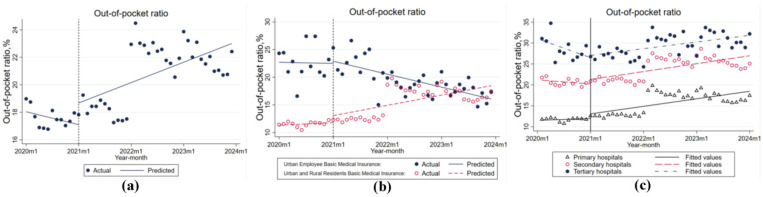
Out-of-pocket ratio. (**a**) Trends of actual and predicted out-of-pocket ratio; (**b**) Comparison of out-of-pocket ratio between Urban Employee Basic Medical Insurance and Urban and Rural Residents Basic Medical Insurance; (**c**) Trends of out-of-pocket ratio across primary, secondary, and tertiary hospitals.

The analysis of the individual out-of-pocket (OOP) percentage, obtained by dividing the patient’s out-of-pocket costs by the total cost of care, revealed different patterns for different healthcare types and hospital levels before and after the implementation of the DIP reform. The average hospital OOP showed a decreasing and then increasing trend, decreasing by 0.001% per month before the reform (*p* > 0.05), increasing by 0.016% in the month after the reform (*p* < 0.05), and showing a significant monthly increasing trend as the policy progressed (0.002%, *p* < 0.01), as shown in Table 4 and Figure 4a.

As shown in Figure 4b, the average OOP level of urban workers was higher than that of urban and rural residents before the reform. After the policy implementation intervention, the individual out-of-pocket ratio of urban workers showed a decreasing trend (−0.002%, *p* > 0.05), in contrast to urban and rural residents, who showed a significant increasing trend (0.001%, *p* < 0.05).

Before the reform, individual out-of-pocket payments for patients in level I and level II hospitals showed a downward trend, while those in level III hospitals showed an upward trend. However, after the implementation of the policy, hospitals at all levels showed an upward trend, with level II hospitals showing the fastest growth and level III hospitals having the highest level of individual out-of-pocket payments overall after the reform. Table 4 and Figure 4c illustrate this trend.

We also examined the 30-day readmission rate as a proxy for care quality; the analysis revealed no statistically significant change following the reform.

## 4. Discussion

This is the first empirical study to compare DIP reform’s effects across health insurance types and hospital levels in a central China pilot city. Based on the health insurance billing data of City M, this study evaluates the effect of the DIP reform on hospitalization costs, hospital days, and individual out-of-pocket payments using an interrupted time series analysis. The DIP reform reduced hospitalization costs and length of stay but increased the out-of-pocket payment ratio. This phenomenon contradicts the objectives of the reform and requires an in-depth analysis of its reasons and corresponding reflections from the perspective of policy optimization. However, it is important to note that the effect sizes associated with these changes were small (Cohen’s d ranging from 0.11 to 0.27), indicating that the statistical significance observed is largely attributable to the large sample size rather than clinically substantial improvements. This underscores the need for cautious interpretation of findings, particularly when considering policy implications.

First, DIP reform aims to control the unreasonable growth of medical costs through the prepayment system. This study found that after the implementation of the DIP payment reform, the total hospitalization cost per patient showed an immediate significant decline of 3.7% at the first month post-reform, followed by a sustained monthly decline of 0.8%, indicating that the reform has achieved some success in cost control [40,41]. However, this cost reduction was mainly concentrated in the immediate post-reform period, and the cost reduction trend gradually weakened over time. This may be related to the gradual adaptation of healthcare organizations to DIP reform policies. In the initial stage of the reform, healthcare institutions may have reduced costs by optimizing internal management and reducing unnecessary tests and treatments. However, over time, the marginal effect of such measures may diminish, which could explain the attenuation of the cost reduction trend. In addition, there are differences in the effect of the DIP reform on different types of hospitals [42]. Hospitalization costs in tertiary hospitals declined more sharply at the beginning of the reform but then rebounded, while hospitalization costs in primary and secondary hospitals continued to decline. This difference may be related to the level of resources and technology in hospitals. Tertiary hospitals usually have more advanced equipment and richer medical resources, and can optimize the treatment process more effectively and reduce unnecessary hospitalization time. In contrast, patients in tertiary hospitals have relatively simple conditions and shorter hospitalization days, with limited room for further reduction [26]. This result is consistent with the study by Liao Zangyi [43]. who found that DRG reforms are more effective in controlling costs in tertiary care hospitals, but that cost rebound may occur in the long run.

Our study also shows that the DIP reform was associated with cost reductions that coincided with shorter hospital stays. This study found that compared to the pre-reform period, the number of hospitalization days showed an immediate decrease of 0.579 days at the policy implementation, and the trend subsequently changed by −0.099 days per month. This indicates improved efficiency. However, there were differences in the effect of different hospital types on hospitalization duration. Tertiary hospitals experienced a greater decline in hospital days after the reform, which may be related to the resource and technological advantages of tertiary hospitals, enabling them to optimize the treatment process more efficiently and reduce unnecessary hospital stays [44,45]. By contrast, the decline in the number of hospitalization days in tertiary hospitals was smaller, which may be related to the fact that patients in tertiary hospitals have relatively simple conditions and shorter hospitalization days, with limited room for further reduction. At the same time, the decline in hospitalization days may also be related to providers’ adaptation to DIP reform [43,46]. In the initial stage of the reform, healthcare institutions may have responded to the economic pressures of the reform by optimizing internal management and improving bed turnover, thus achieving a significant decline in the number of inpatient days. However, over time, some healthcare organizations may gradually explore ways to increase revenue without increasing the number of inpatient days [47,48,49,50], resulting in a gradual decrease in the trend of declining inpatient days. Similar patterns have been observed in DRG reform studies, which report initial cost reductions followed by potential rebound, as well as short-term reductions in hospital days with subsequent slowing of the downward trend [43,44]. While total costs decreased (¥6015 to ¥5138), both absolute OOP payments (¥1013 to ¥1084) and their share of total costs (17.7% to 21.2%) increased. This pattern suggests that patients bore a larger share of a smaller total. As shown in Table 1 and Table 4, and Figure 3b, the increase in the individual out-of-pocket ratio after the urban and rural residents’ health insurance reform may reflect that the decrease in patients’ out-of-pocket costs was proportionally smaller than the decrease in total medical costs, which leads to the overall individual out-of-pocket ratio showing an upward trend. This phenomenon may be related to the following factors. Changes in cost structure: Although the DIP reform has reduced total hospitalization costs, it may not have effectively controlled the absolute value of out-of-pocket expenses [51,52,53]. One interpretation is that providers shifted costs to patients by adjusting the cost structure. For example, some drugs and examination items not on the medical insurance list may be used more frequently, increasing the out-of-pocket burden of patients. Adjustment of medical services: Hospitals may reduce non-essential services to cut costs, shifting rehabilitation or treatment expenses to patients [27,45]. Changes in patient selectivity: DIP reform may have affected patients’ choice of care. Some patients may choose fewer medical services for fear of increased hospitalization costs or may choose to obtain medical services from other sources, such as outpatient clinics, resulting in a relative increase in out-of-pocket costs during hospitalization [53]. Adjustment of health insurance reimbursement policies: DIP reform may be accompanied by adjustments in health insurance reimbursement policies, such as raising the threshold and lowering the reimbursement rate. These policy changes may have contributed to the observed increase in patients’ out-of-pocket expenses [54].

Taken together, these factors represent potential explanations for the observed increase in out-of-pocket payments, though the precise contribution of each cannot be definitively determined from our data.

Furthermore, the effectiveness of DIP reform is inherently shaped by the governance framework within which it operates, as outlined in the Introduction. While national guidelines provide a standardized architecture, local adaptations—such as the setting of payment coefficients, supervision intensity, and budget allocation accuracy—may introduce heterogeneity in implementation across regions and institutions [15,16,17,18]. This governance heterogeneity could partly explain the differential effects observed across hospital levels in our study: tertiary hospitals, with greater administrative capacity and closer alignment with local health authorities, might adapt more effectively to the new payment incentives, whereas primary and secondary hospitals may face greater challenges in implementation, potentially leading to unintended consequences such as cost shifting or patient selection. Moreover, disparities in local governance quality could exacerbate inequities in patient outcomes, particularly if weaker governance fails to adequately monitor provider behavior or protect vulnerable populations. Although our study could not directly measure governance characteristics due to data limitations, these considerations underscore the need for future research to examine how variations in governance shape the equity and efficiency of DIP reform, and to inform targeted strategies for strengthening institutional capacity, especially in under-resourced settings. Unmeasured clinical severity and socioeconomic factors may bias our estimates. If post-reform patients were systematically sicker, cost reductions could be underestimated; if healthier, overestimated. Thus, our findings should be interpreted as associations rather than definitive causal effects.

## 5. Conclusions

This ITS analysis evaluated DIP reform’s effects on costs, length of stay, and OOP payments in M City. The DIP reform was associated with reductions in hospitalization costs and hospital days, alongside an increase in out-of-pocket payments. This phenomenon may be related to factors such as changes in the cost structure, adjustments in medical services, changes in patient selectivity, and adjustments in health insurance reimbursement policies. This increased financial burden on patients raises concerns about potential downstream effects on healthcare access, treatment adherence, and long-term health outcomes—underscoring the importance of monitoring not only costs but also patient-centered metrics such as readmissions, exacerbations, and mortality. Future efforts should optimize payment rules, strengthen supervision, and refine reimbursement policies to better achieve reform objectives, reduce patient burden, and improve care quality.

DIP reform, as an important direction in China’s health insurance payment method reform, has important policy significance. However, the implementation effect of the reform must be evaluated in multiple dimensions, including medical costs, medical service efficiency, and medical service quality. The DIP reform was associated with short-term reductions in costs and length of stay, though unmeasured confounding warrants cautious interpretation. In addition, an increase in individual out-of-pocket payments may undermine the actual benefits of the reform for patients. This suggests that DIP reform needs to strike a better balance between cost containment and patient burden.

Based on these findings, several policy recommendations emerge. First, given the observed increase in out-of-pocket payments despite overall cost reductions, policymakers should strengthen oversight of service composition to prevent cost shifting from insurers to patients. This could include monitoring the use of non-covered drugs and procedures, and adjusting reimbursement lists to align with clinical necessity. Second, the differential effects across hospital levels suggest that tailored implementation strategies may be warranted: tertiary hospitals may require additional guidance to sustain initial cost reductions, while primary and secondary hospitals could benefit from sharing best practices in efficiency improvement. Third, the rising OOP ratio highlights the need for complementary policies to protect vulnerable patients, such as targeted subsidies or expanded coverage for essential medications, to ensure that cost containment does not compromise equitable access to care.

Our findings align with recent studies on DIP reform in China, which have similarly documented reductions in hospitalization costs and length of stay, alongside increased out-of-pocket payments [12,44,45]. The heterogeneous effects across hospital levels observed in our study are also consistent with prior research [27,52]. This convergence with existing evidence strengthens confidence in our results and suggests that the patterns we observed reflect broader dynamics of DIP reform rather than being idiosyncratic to M City.

## 6. Limitations and Future Research Directions

### Strengths and Limitations of This Study

First multilevel comparative evaluation: This study provides the first empirical assessment of the differential effects of the DIP payment reform across hospital levels (primary, secondary, tertiary) and major health insurance schemes (URRMI vs. UEBMI) within a real-world pilot setting, offering granular insights for policy refinement.

Methodological strength: The application of interrupted time series analysis to 48 months of longitudinal, city-wide claims data allows for a rigorous quasi-experimental evaluation, effectively distinguishing the reform’s immediate and sustained effects from underlying temporal trends.

Limited generalizability and unobserved confounders: The single-city, observational design limits the external validity of findings. Furthermore, the absence of a control group and detailed patient-level clinical/comorbidity data restricts the ability to fully isolate the reform’s effect from concurrent events (e.g., COVID-19); additionally, the universal and simultaneous implementation of DIP in M City precluded the use of a difference-in-differences design with an internal control group. Lack of clinical and quality outcomes: Reliance on administrative claims data precludes analysis of key clinical metrics (e.g., COPD severity, lung function, readmission rates) and direct measures of healthcare quality; the study cannot assess whether cost and length-of-stay reductions were achieved without compromising patient health outcomes. We acknowledge the absence of more granular clinical severity measures, such as ICU admission rates, mechanical ventilation usage, or other disease-specific clinical endpoints. These data are unavailable in administrative insurance databases, and their inclusion would facilitate a more comprehensive assessment of the reform’s effect on patient health outcomes. Unmeasured confounders—including clinical severity, comorbidity burden, and socioeconomic factors—may bias our estimates and limit causal inference.

Short-term evaluation window: The post-reform observation period (approximately 3 years) may be insufficient to capture long-term adaptations by healthcare providers, potential cost rebounds, or the full evolution of the policy’s effect on patient financial burden and system efficiency.

Future studies could further explore the effect of DIP reform on different types of diseases and different patient groups, as well as the long-term effect of the reform on the quality of healthcare services. In addition, this study can consider setting up a control group to more accurately assess the effect of the reform. These studies can provide a scientific basis for further optimization of DIP reform and promote the continuous development of China’s health insurance payment method reform.

Finally, this study focuses on patient-level outcomes without examining the broader health financing context, including the balance between private and public sources, linkages to government revenue (e.g., taxation), and implications for optimal budget allocation and social balance. These macro-level dynamics, while beyond our scope, represent important directions for future research on how DIP reform interacts with fiscal sustainability and social welfare.

## Data Availability

The data presented in this study are available on request from the corresponding author. Because the data were obtained from the Hubei Provincial Medical Insurance Information Platform database, the datasets generated or analyzed in this study are not publicly available.

## References

[1] Soriano J.B., Polverino F., Cosio B.G. (2018). What Is Early COPD and Why Is It Important?. Eur. Respir. J..

[2] Bagdonas E., Raudoniute J., Bruzauskaite I., Aldonyte R. (2015). Novel Aspects of Pathogenesis and Regeneration Mechanisms in COPD. Int. J. Chronic Obstr. Pulm. Dis..

[3] Gan L., He X., Wu J. (2025). Impact of Moderate and Severe Exacerbations on Clinical Prognosis and Economic Burden of Chronic Obstructive Pulmonary Disease in China. Expert Rev. Pharmacoecon. Outcomes Res..

[4] Dou L., Zheng Y., Feng J., Huang Z., Qin F., Gao M., Li S. (2025). The Humanistic and Economic Burden of COPD Patients in Urban China: A Propensity Score Matching Study. Int. J. Chronic Obstr. Pulm. Dis..

[5] Yang T., Wu J., Chi C. (2025). Investing in Primary Care to Reduce the Burden of Chronic Obstructive Pulmonary Disease. China CDC Wkly..

[6] Barrett R., Barrett R. (2021). Asthma and COPD Medicines Prescription-Claims: A Time-Series Analysis of England’s National Prescriptions during the COVID-19 Pandemic (Jan 2019 to Oct 2020). Expert Rev. Respir. Med..

[7] Negewo N.A., Gibson P.G., McDonald V.M. (2015). COPD and Its Comorbidities: Impact, Measurement and Mechanisms. Respirology.

[8] Agustí A., Vogelmeier C., Faner R. (2020). COPD 2020: Changes and Challenges. Am. J. Physiol.-Lung Cell. Mol. Physiol..

[9] Polverino F., Sam A., Guerra S. (2019). COPD: To Be or Not to Be, That Is the Question. Am. J. Med..

[10] Halpin D.M.G. (2024). Mortality of Patients with COPD. Expert Rev. Respir. Med..

[11] López-Campos J.L., Tan W., Soriano J.B. (2016). Global Burden of COPD. Respirology.

[12] Wang Y., Huang X., Lu N., Xu X., Wang L., Du W., Xu W. (2025). The Effects of the Diagnosis-Intervention Packet Payment Reform in China: Evidence from Guangzhou. Front. Public Health.

[13] Wu L., Wu N., Cao Y., Zhou X. (2025). The Implementation Effect of DIP Payment Method across Different Population in Southwest China Based on Multi-Group Interrupt Time Series. Front. Public Health.

[14] Jin Z.-Q., Li Y.-L., Tao Y.-Y., Shen K.-Y., Lin X.-H., Pei T., Li C.-C., Wu D., Meng X.-H. (2025). Impact of Diagnosis-Intervention Packet Payment on the Consistency of Hospitalization Expenses across Different Medical Insurance Schemes in China. Front. Public Health.

[15] China’s NHSA Issued the Regulations on DRG/DIP Healthcare Security Payment Mechanism. https://cms-lawnow.com/en/ealerts/2025/09/china-s-nhsa-issued-the-regulations-on-drg-dip-healthcare-security-payment-mechanism.

[16] Chang J., Chen S., Li A., Yang X., Luo H., Yilamu M., Fu B., Xu N., Liu J., Tian H. (2025). Facilitators and Barriers to the Implementation of DIP Payment Methodology Reform in a Public Hospital in Guangzhou: A Qualitative Study Based on the Implementation of the Meta-Framework for Research (CFIR) Framework. Front. Public Health.

[17] Journal of China Health Insurance Published: Jiujiang’s “Medical Insurance Data Hub” Unlocks New Value in Fund Governance. Jiujiang Municipal Healthcare Security Bureau [Official Website], Work Updates, Municipal Level. https://ybj.jiujiang.gov.cn/zwzx_209/gzdt/sjdt/202511/t20251126_7093406.html.

[18] Yao Q., Zhang X., Yao L. (2024). The Settlement Mechanism of Diagnosis-Intervention Packet Payment Scheme in China: A Policy Review and Lessons Learned. Inform. Health.

[19] Su D., Chen Y., Gao H., Li H., Chang J., Lei S., Jiang D., Hu X., Tan M., Chen Z. (2019). Is There a Difference in the Utilisation of Inpatient Services between Two Typical Payment Methods of Health Insurance? Evidence from the New Rural Cooperative Medical Scheme in China. Int. J. Environ. Res. Public Health.

[20] Li Y., Lu C., Liu Y. (2020). Medical Insurance Information Systems in China: Mixed Methods Study. JMIR Med. Inform..

[21] Stevens S. (2015). Preventing 30-Day Readmissions. Nurs. Clin. N. Am..

[22] Luo J., Wang S., Dan L., Zhang R., Bi L. (2022). Research on the Influence of Payment Methods on the Control of Medical Insurance Expenses-Based on Empirical Analysis of Double Difference. Front. Public Health.

[23] Li L., Yu Q. (2024). The Impact of Reform of Medical Insurance Payment Method on Medical Service Pricing-Based on Empirical Analysis of Matched Medical Finance Comprehensive Data. BMC Health Serv. Res..

[24] Andersen R.M. (1995). Revisiting the behavioral model and access to medical care: Does it matter?. J. Health Soc. Behav..

[25] Donabedian A. (1988). The quality of care. How can it be assessed?. JAMA.

[26] Zhang Y., Xu S.-Y., Tan G.-M. (2024). Unraveling the Effects of DIP Payment Reform on Inpatient Healthcare: Insights into Impacts and Challenges. BMC Health Serv. Res..

[27] Tang X., Zhang X., Chen Y., Yan J., Qian M., Ying X. (2023). Variations in the Impact of the New Case-Based Payment Reform on Medical Costs, Length of Stay, and Quality across Different Hospitals in China: An Interrupted Time Series Analysis. BMC Health Serv. Res..

[28] Wagner A.K., Soumerai S.B., Zhang F., Ross-Degnan D. (2002). Segmented regression analysis of interrupted time series studies in medication use research. J. Clin. Pharm. Ther..

[29] Bernal J.L., Cummins S., Gasparrini A. (2017). Interrupted time series regression for the evaluation of public health interventions: A tutorial. Int. J. Epidemiol..

[30] Kontopantelis E., Doran T., Springate D.A., Buchan I., Reeves D. (2015). Regression Based Quasi-Experimental Approach When Randomisation Is Not an Option: Interrupted Time Series Analysis. BMJ.

[31] Austin P.C. (2011). An Introduction to Propensity Score Methods for Reducing the Effects of Confounding in Observational Studies. Multivar. Behav. Res..

[32] Rosenbaum P.R., Rubin D.B. (1983). The central role of the propensity score in observational studies for causal effects. Biometrika.

[33] Mercado N., Ito K., Barnes P.J. (2015). Accelerated Ageing of the Lung in COPD: New Concepts. Thorax.

[34] Calle Rubio M., Esmaili S., Esmaili I., Gómez Martín-Caro L., Ayat Ortiz S., Rodríguez Hermosa J.L. (2025). Sex-Based Disparities in Clinical Burden and Diagnostic Delay in COPD: Insights from Primary Care. J. Clin. Med..

[35] Choate R., Aksamit T.R., Torrence J., DiLorenzo P.A., Rodriguez A., Miller B., Wright J., DeMeo D.L. (2025). Navigating COPD and Bronchiectasis: A COPD Foundation Survey of Differences in Patient-Perceived Health Care Experiences by Sex. Chronic Obstr. Pulm. Dis..

[36] Manning W.G., Mullahy J. (2001). Estimating Log Models: To Transform or Not to Transform?. J. Health Econ..

[37] Wang J., Li P., Wen J. (2020). Impacts of the Zero Mark-up Drug Policy on Hospitalization Expenses of COPD Inpatients in Sichuan Province, Western China: An Interrupted Time Series Analysis. BMC Health Serv. Res..

[38] Li P., Duan Z., Zhang Z., He Y., Li W., Wen J. (2020). Impacts of Government Supervision on Hospitalization Costs for Inpatients with COPD: An Interrupted Time Series Study. Medicine.

[39] Bottomley C., Ooko M., Gasparrini A., Keogh R.H. (2023). In Praise of Prais-Winsten: An Evaluation of Methods Used to Account for Autocorrelation in Interrupted Time Series. Stat. Med..

[40] Newey W.K., West K.D. (1987). A Simple, Positive Semi-Definite, Heteroskedasticity and Autocorrelation Consistent Covariance Matrix. Econometrica.

[41] Shi T., Meng L., Li D., Jin N., Zhao X., Zhang X., Liu Y., Zheng H., Zhao X., Li J. (2022). Impact of the Expanded Program on Immunization on the Incidence of Japanese Encephalitis in Different Regions of Mainland China: An Interrupt Time Series Analysis. Acta Trop..

[42] Okuno T., Takada D., Shin J.-H., Morishita T., Itoshima H., Kunisawa S., Imanaka Y. (2021). Surgical Volume Reduction and the Announcement of Triage during the 1st Wave of the COVID-19 Pandemic in Japan: A Cohort Study Using an Interrupted Time Series Analysis. Surg. Today.

[43] Chen Y.-J., Zhang X.-Y., Tang X., Yan J.-Q., Qian M.-C., Ying X.-H. (2023). How Do Inpatients’ Costs, Length of Stay, and Quality of Care Vary across Age Groups after a New Case-Based Payment Reform in China? An Interrupted Time Series Analysis. BMC Health Serv. Res..

[44] Ding Y., Yin J., Zheng C., Dixon S., Sun Q. (2023). The Impacts of Diagnosis-Intervention Packet Payment on the Providers’ Behavior of Inpatient Care-Evidence from a National Pilot City in China. Front. Public Health.

[45] Qian M., Zhang X., Chen Y., Xu S., Ying X. (2021). The Pilot of a New Patient Classification-Based Payment System in China: The Impact on Costs, Length of Stay and Quality. Soc. Sci. Med..

[46] Liao C., Jing O., Hui W. (2024). Medical Service Value Enhanced by Medicare DRG Payment Reform: Evidence from H City. Res. Financ. Econ. Issues.

[47] Lin K., Yao Y., Xiong Y., Xiang L. (2024). The Effect of an Innovative Payment Method on Inpatient Volume and Bed Resources and Their Regional Distribution: The Case of a Central Province in China. Int. J. Equity Health.

[48] Yan J., Shi Y., Zhang J., Chen S., Huo X., Shen Y., Zhang N. (2023). Impact of Capitation Prepayment on the Medical Expenses and Health Service Utilization of Patients with Coronary Heart Disease: A Community Policy Intervention Program in a County in China. BMC Public Health.

[49] Wen L., Divers C., Lingohr-Smith M., Lin J., Ramsey S. (2018). Improving Quality of Care in Oncology through Healthcare Payment Reform. Am. J. Manag. Care.

[50] Lin K., Li Y., Yao Y., Xiong Y., Xiang L. (2024). The Impact of an Innovative Payment Method on Medical Expenditure, Efficiency, and Quality for Inpatients with Different Types of Medical Insurance: Evidence from a Pilot City, China. Int. J. Equity Health.

[51] Yang F., Chen M., Si L. (2022). What Can We Learn from China’s Health Insurance Reform to Improve the Horizontal Equity of Healthcare Financing?. Int. J. Equity Health.

[52] Zhu C., Li Z., Lin F., Huang L., Cao K., Xiao Y., Yang J., Zhu J., Li H., Li W. (2025). The Impact of Diagnosis-Intervention Packet (DIP) Payment on Cost Structure of Inpatient Care—Evidence from a Tertiary Hospital in China. PLoS ONE.

[53] Wang Y., Liu S., Zhang X., Ma H., Ying X. (2025). Impact of an Innovative Case-Based Payment Reform on Hospital Cost Variation: Insights from Cerebral Infarction Inpatients in China. Int. J. Equity Health.

[54] Shen K., Tao Y., Li Y., Jin Z., Li C., Wu D., Meng X. (2025). Impact of Diagnosis-Intervention Packet (DIP) Reforms on Inpatient Services for Low-Income Populations in Central China: A Multi-Stage Interrupted Time-Series Analysis. PLoS ONE.

